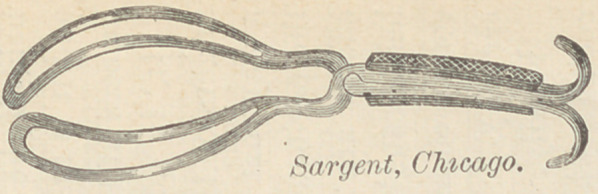# How to Save the Perineum

**Published:** 1878-05

**Authors:** Edw. Warren Sawyer

**Affiliations:** Lecturer on Obstetrics and Diseases of Children, Rush Medical College, Chicago


### HOW TO SAVE THE PERINEUM.—A NEW USE OF THE OBSTETRIC FORCEPS.—AN IMPROVED INSTRUMENT.

(*Remarks made before the Chicago Society of Physicians and Surgeons, April* 8, 1878.)

By Edw. Warren Sawyer, M. D.,

Lecturer on Obstetrics and Diseases of Children, Rush Medical College, Chicago.

Mr. President and Gentlemen:—The remarks which I have to offer, relate to those labors in which the vertex is in advance, and only to that stage of labor when the head has almost passed through the bony portion of the obstetric canal, but is still opposed chiefly by the woman's soft parts at the floor of the pelvis, and the outlet of the canal.

It will perhaps elucidate the point I hope to establish, if I may be permitted to describe the manner in which the forces of the woman will complete the expulsion of the head, if no interference is offered by the attendant.

At the moment we speak of, the antero-posterior diameter of the fœtal head approximately corresponds to the same diameter of the parturient canal. In the great preponderance of labors, the back of the head looks upward—the woman being upon the back. A little further advance brings the vertex to look through the vulvar aperture, and the nape of the neck in contact with the inner surface of the pubes; while that part of the occiput just inferior to the protuberance, is lodged against the sides of the pubic arch. The occiput is too broad to be received into the pubic arch as far as its summit, as one may convince himself by sweeping the tip of the finger between the inferior border of the symphysis and the head, during the expulsion of the latter.

Till this time the fœtal head has been in a state of flexion; but, when the occipital plane becomes arrested against the pubic arch, the frontal part of the head receives the propelling force more directly, and is soon in advance. The head is now made to describe a movement of extension, or evolution, by which it becomes unfolded into the world, around the point of the pubic arch, against which the occiput is lodged, as around a pivot.

Two forces operate to produce this extension. The propelling power of the uterus, and its auxiliaries, the *vis-a-tergo*, advance the head until the larger portion of the forehead looks over the margin of the perineum. When the soft-parts become still further distended by the fœtal head, the perineum draws itself backward over the face, urging forward successively the margin of the orbit, the malar prominences, nose, lips and chin, the part of the head to be freed last being that part which was lodged against the pubic arch. This retraction of the perineum is the second force which extends the head.

Such is an outline of the manner in which the head is delivered by nature. A movement which, while it succeeds in its object, at the same time jeopardizes the woman's soft-parts. This is even more apparent when we recall the successively increasing dimensions of the head which pass through the vulvar outlet. I have given the name pubo-facial diameter to that axis, one end of which rests upon the highest part of the pubic arch, while the opposite extremity is lost upon different parts of the face. Their names indicate the limits more exactly. The length of these several axes show the extent to which the vulvar aperture is opened to give exit to the head. Thus the pubo-frontal, $4\frac{1}{2}$ inches; pubo-malar, or nasal, $4\frac{3}{4}$ inches; pubo-mental, $5\frac{1}{4}$ to $5\frac{1}{2}$ inches. I believe it practically impossible for the vulva of the primiparous woman to be stretched to this degree without a rupture of the perineum occurring. I am aware that a primipara may be delivered of a large child and her perineum be left intact, but this is because the judicious interference of her attendant compelled the head to pass out in a shorter axis than nature can do if left to herself.

I have had an opportunity of an occular demonstration of the moment and manner in which the perineum was torn. Just as the upper margin of the orbits looked over the edge of the perineum, the little fold of mucous membrane known as the fourchette, or frænum, gave way; this tear was continuously deepened by the malar prominences and chin. This is, I think, the usual order. Writers have described, in exceptional cases, its first giving way at the center. But all agree that it tears on the median line.

I know some hold that it is the bis-acromial diameter, the shoulders, which causes the tear. But I cannot believe that the perineum left absolutely intact by the head, will be torn first by the shoulders, under the care which the woman always receives. Such an accident can always be averted by delivering the pubic shoulder first.

The interference which I would recommend, to anticipate the hazardous stretching of the vulva, is, in a word, of a nature to hold the head in a state of extreme flexion, and force it to pass through the vulva in a diameter a little superior to the pubo-frontal, and which has a length in the full grown child of about four inches.

I cannot assume that preventing the extension of the head at this time is an original procedure; only that its significance is not generally understood. Many practitioners interfere during this stage of labor, and really prevent the complete extension of the head. Thus, some hook the index finger over the chin through the woman's rectum. It would seem, at first sight, that they attempted just what we would prevent, that is, extension of the head; but really they accomplish what we have recommended; for the thumb of the same hand is pressed upon the perineum which is being bulged out by the forehead. In this way, with the face held between the thumb and finger, in easy cases, the sinciput is kept back.

Others apply the hand to the perineum in such a way that the commissure between the thumb and finger corresponds with the posterior commissure of the vulva. Judicious support, continuously supplied, is of the greatest advantage; but the object to be attained by this palmar pressure is not always understood; for I have often been told to hold the forehead back for a time to allow the perineum to become thin, and the vulva more easily stretched. Possibly this is accomplished; but the greatest advantage comes from the flexion into which the head has, by this means, been forced.

In addition to holding the sinciput back with one hand, others attempt to tease the occiput forward, with the fingers of the other hand applied to the head just in front of the symphysis pubis.

Besides the objections to the introduction of the finger in the rectum, it can be said against all these measures, that they are not sufficient, in the majority of labors, to hold the head in a state of flexion.

I am assured that this can be most certainly and easily accomplished with the forceps. For a long time I have been using my short forceps for this purpose, with the most satisfactory results.

I have recently modified my forceps to make it more easily used for this purpose, viz: I have continued the pelvic curve forwards to the handle, giving the entire instrument a regular curve from the extremity of the blade to the end of the handle.* This curve assists the operator in giving a proper direction to his traction, and makes the instrument most useful for the operation which I will detail presently, at the same time that the general usefulness of the instrument as a short forceps, is enhanced.

Length of instrument, 10 inches; length of blade to lock, $6\frac{1}{2}$ inches; length of chord of head curve, $5\frac{3}{4}$ inches; widest part of blade, $1\frac{1}{2}$ inches; greatest distance between blades, $2\frac{3}{4}$ inches; distance between tip of blades, $\frac{1}{2}$ inch; weight, 7 oz.

NOTE.—The handle is to be seized in such a manner, that the palm of the hand looks upward; the hook of one blade will then rest naturally upon the lateral and extensor surface of the first phalanx of the index finger, and the other hook upon the corresponding part of the thumb.

* I have also added this curve to the long forceps; which, besides guiding the operator in making traction always in the right axis, is peculiarly adapted to those high operations where the perineum and coccyx do not allow the instrument to be directed backward to a sufficient degree.

Let us assume that the forceps is to be used to flex the head, and to hold it in a state of flexion. At this stage in the labor the woman is usually upon the back. Her position need not be changed, only to have the limbs strongly flexed, in the lithotomy position. Nor is it necessary for the operator's body to be in a line with the pelvic axis. The introduction and locking of the blades, is a matter of extreme simplicity. While the head is loosely held, the handles should be elevated, so as to nearly approach the symphysis. Choose the interval of uterine action. Now clasp the head firmly in the blades, and slowly depress the handles until the edge of the perineum is approached. It may be that one movement is not sufficient to flex the head; this is the more likely to result if the operator is overtaken by a contraction of the uterus. It is only necessary, in this event, to repeat the elevation and depression of the handles, and the operator will have the satisfaction of feeling the sinciput recede, when a moment before it was causing the perineum to bulge out in a most threatening manner.

The most important step in the operation remains to be mentioned. When the head has been flexed, the hold of the instrument should be relaxed, and the handles elevated to that degree that the general axis of the handle is nearly perpendicular to the plane of the bed. It is in this position that the head is to be held, if the operator waits for the uterus to complete the delivery; and it is in this direction only that the operator lifts the head, if he sees fit to make traction.

If, in addition to this use of the forceps, the ends of the fingers of the disengaged hand are placed upon the head, in such a way that the convex surfaces of the nails rest upon the edge of the perineum, and, during the interval of uterine action, gentle efforts are made to tease back this edge, and to prevent it from being caught upon the advancing head, the operator has, by this conjoined manipulation, given the soft parts the greatest possible security.

Finally, in an exceptional case of posterior position of the occiput, which refused to rotate forward to appear beneath the pubic arch, I was able, by reversing the movement I have described, to lift the head into marked flexion, and to deliver a primipara of a large child without injury to the soft parts.

As injuries of the soft parts, I have, in the foregoing remarks, alluded particularly to those rents which are apparent upon an inspection of the perineum. But, it seems reasonable that the liability to those not infrequent tears of the mucous membrane about the outlet, would be lessened by the same measures which would prevent the dangerous distension of the perineum.

## Figures and Tables

**Figure f1:**
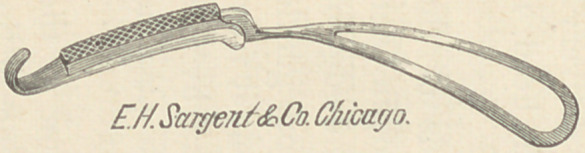


**Figure f2:**